# Vitamin D status of the Russian adult population from 2013 to 2018

**DOI:** 10.1038/s41598-022-21221-4

**Published:** 2022-10-05

**Authors:** Daria V. Smirnova, Colin D. Rehm, Ronald D. Fritz, Inga S. Kutepova, Maria S. Soshina, Yulia A. Berezhnaya

**Affiliations:** 1 PepsiCo R&D, Leningradsky prospect 72 k4, 125319 Moscow, Russia; 2 PepsiCo R&D, Purchase, NY USA

**Keywords:** Calcium and vitamin D, Epidemiology, Risk factors

## Abstract

Vitamin D deficiency is widespread globally, however available data for the Russian adult population is fragmented. This cross-sectional study used secondary data for individuals undergoing testing for vitamin D concentrations from 2013 to 2018 by InVitro laboratory. 25(OH)D serum concentration was determined using chemiluminescent microparticle immunoassay. The mean, median, and proportion with severe, deficient, insufficient and sufficient 25-hydroxyvitamin D (25(OH)D) concentrations were estimated. Splines examined the effect of latitude on 25(OH)D concentrations. Data were available for 30,040 subjects age ≥ 18 years. 24.2% of the sampled population had sufficient (30–< 150 25(OH)D ng/mL), 34% deficient (10–19.9 ng/mL) and 5.6% severely deficient (< 10 ng/mL) status. Average 25(OH)D concentrations were highest among 30–44 years and lowest amongst older adults; females had modestly higher values. Concentrations were 15% higher in fall/summer vs. winter/spring. A non-linear relationship was observed by latitude; the highest 25(OH)D concentrations were observed near 54°N, decreasing at more southern latitudes for women and more northern latitudes for both sexes. These results are comparable to other Northern European publications and limited Russian samples demonstrating low concentrations. Acknowledging that nationally-representative and randomly sampled data are needed, the present data suggest the burden may be high and identifies some population sub-groups and geographic areas with a higher potential deficiency of vitamin D.

## Introduction

Vitamin D is naturally produced by human skin under UVB light exposure as well as consumed from dietary sources including oily fish, fortified or enriched products, typically milk and dairy and dietary supplements. After several chemical modifications of vitamin D in the human body its metabolite 1,25 (OH)D interacts with vitamin D receptors, which directly or indirectly regulate expression with more than 2000 genes^1–3^. This results in a wide range of biological actions not limited by calcium and phosphorus exchange and bone mineralization. Because of the variety of its functions along with growing concern about health risks associated with low vitamin D status, there are debates among different medical and public health authorities on what a sufficient or optimal concentration of this vitamin is, as estimated by its the most stable metabolite 25(OH)D concentration in serum^4^. This has led to further interest regarding the adequate supply of this vitamin to humans^5^.

There are many studies related to identification of main risk factors for vitamin D insufficiency, status evaluation, and strategies for its improvement globally. For European countries, several meta-analyses are available^6^ and include data from more than 600,000 healthy participants extracted from more than 100 studies^7^, with sub-national data for many countries. These show widespread prevalence of low intake of vitamin D, and therefore deficiency or insufficient status among the resident population for most countries assessed. Several international projects such as OptiFord^8^ (2000–2007, 5 countries: Finland, Denmark, Ireland, Spain, Poland) and more recent ODIN^9^ (2013–2018, 19 countries: Ireland (lead), the United Kingdom, Spain, Portugal, Norway, Denmark, Iceland, Finland, Austria, Germany, Slovakia, Serbia, Switzerland, Greece, the United States, Ukraine, Belgium, the Netherlands and Canada) were conducted with the aim to find food-based solutions for optimal vitamin D nutrition and health through the life cycle. Trends in vitamin D status appear to be quite heterogenous, with improvements observed in many countries/sub-populations (namely those implementing fortification programs or promoting widespread supplementation), while progress has not been made for other populations, namely those who may be undergoing transitions away from a traditional diet^10^.

Additionally, there have been efforts to standardize the measurement of circulating 25(OH)D with the aim to achieve comparability of results obtained in different time points, laboratories, and via different assays. The Vitamin D Standardization Program (VDSP) organized by the Office of Dietary Supplements of the National Institutes of Health (USA), was established in 2010 to address this issue^11,12^. The VDSP protocols for standardization of serum 25(OH)D data from past surveys have been applied to national surveys in Canada^13^, the US^14^ and numerous nationally or regionally representative samples in Europe (Danish, 2006; Norwegian, 2000; Finland, 2011^15^; German^16^).

Like other northern European countries, the Russian population is at potentially high-risk of vitamin D deficiency due to insufficient sun exposure (i.e., the low efficiency of its endogenous synthesis in the skin), as well as infrequent consumption of the main source of oil-rich fish and few vitamin D fortified products (e.g., dairy foods). Despite possessing a high risk of vitamin D deficiency, the status for the general Russian population is lacking due to the absence of a national vitamin D monitoring program which is complicated by Russia’s expansive size, different levels of insolation, and ethnic heterogeneity. Existing data on vitamin D status among the Russian population is available for small populations of limited generalizability, due to a recent monograph edited by Kodentsova^17^, epidemiological studies which run in frame of dietary intake monitoring from 2012 to 2017 represented by several groups: children and adolescents (7–14 years; n = 790), students (Arkhangelsk; n = 58), pregnant female (Moscow; n = 100), etc. Despite these limitations, findings are generally consistent in that 50–91% of the working-age population has reduced vitamin D concentrations (< 30 ng/mL) regardless of age, sex and season of data collection^18^. Systematic assessments of the indigenous population of the Russia north also exhibit high levels of sub-optimal vitamin D concentrations^19^. Lastly, inter-laboratory and inter-assay variability can affect estimation of vitamin D status on a population level and thereby potentially adversely influence recommendations for its improvement^20–26^.

Given the lack of data on vitamin D status of the Russian adult population, an ad hoc analysis of a large dataset, obtained with the same assay and extracted from a commercial laboratory, covering all of Russia, has potential to provide new insights into the vitamin D status of the Russian adult population. Further, this data can provide understanding regarding potential associations between vitamin D status and different factors like season, age, sex and location, as well as examine temporal trends, which could be used for risk stratification.

## Results

Among the overall sample size of 30,040 individuals in the dataset, 83% were female, the median age was 53 years and the mean age was 51.8 (standard deviation: 15.5). Subjects were disproportionately more likely to have data taken in the winter/spring (61.6%) as compared to summer/fall (38.4%) and there was a steady increase in the number of tests conducted over time (12% from 2013 to 2014 and 34.3% from 2018). Forty-six percent of subjects lived in the Central Federal District and there were more than 1,000 subjects from each Federal District. Population characteristics are shown in Table 1.

**Table 1:** Multivariable adjusted^a^ mean 25(OH)D concentrations and status overall and by population sub-group in Russia (n = 30,040), 2013–2018.

	N (% of total)	Mean, ng/mL	Median ng/mL (IQR)	Severely deficient (< 10 ng/mL), %	Deficient (10–19.9 ng/mL), %	Insufficient (20–29.9 ng/mL), %	Sufficient (30–149.9 ng/mL), %
Total	30,040	23.9 (23.7, 24.1)	21.7 (16.1, 28.6)	4.4 (4.1, 4.7)	35.3 (34.6, 36.1)	36.5 (35.8, 37.2)	23.8 (23.1, 24.4)
**Age group, y**							
18–29	2,478 (8.2)	22.9 (22.4, 23.5)	20.9 (14.9, 27.9)	6.3 (5.3, 7.2)	37.9 (35.9, 39.9)	34.0 (32, 35.9)	21.9 (20.2, 23.5)
30–44	7,520 (25)	25.1 (24.8, 25.4)	22.5 (17, 29.9)	3.5 (3.1, 3.9)	32.8 (31.6, 33.9)	36.7 (35.5, 37.9)	27 (25.9, 28.1)
45–59	9,936 (33.1)	24.1 (23.8, 24.4)	22.4 (16.6, 28.9)	3.8 (3.4, 4.2)	33.3 (32.2, 34.3)	38.8 (37.7, 39.9)	24.1 (23.2, 25.1)
60–74	7,990 (26.6)	23.4 (23.1, 23.8)	21.4 (15.7, 27.9)	4.5 (4, 4.9)	36.9 (35.6, 38.1)	36.1 (34.9, 37.3)	22.6 (21.6, 23.6)
≥ 75	2,116 (7.0)	21.0 (20.4, 21.5)	18.4 (12.7, 25.9)	11.2 (9.8, 12.7)	43.6 (41.3, 45.8)	28.0 (26, 29.9)	17.2 (15.6, 18.9)
P-trend		< 0.001	< 0.001	< 0.001	< 0.001	0.006	< 0.001
**Sex**							
Female	24,931 (83.0)	24.3 (24.1, 24.4)	22.0 (16.3, 29.1)	4.1 (3.8, 4.3)	33.6 (33, 34.2)	37.6 (37, 38.2)	24.7 (24.1, 25.3)
Male	5,109 (17.0)	23.4 (23.1, 23.8)	21.4 (15.7, 28.1)	4.8 (4.2, 5.3)	37.3 (35.9, 38.7)	35.2 (33.8, 36.6)	22.7 (21.5, 23.9)
P-difference		< 0.001	< 0.001	0.03	< 0.001	0.003	0.004
**Season**							
Summer/fall	11,543 (38.4)	25.9 (25.7, 26.2)	23.9 (18, 31.1)	2.6 (2.3, 2.9)	27.7 (26.8, 28.6)	39.7 (38.7, 40.7)	30.0 (29, 30.9)
Winter/spring	18,497 (61.6)	22.6 (22.4, 22.8)	20.4 (14.8, 27.1)	5.9 (5.5, 6.3)	40.1 (39.3, 41)	33.9 (33, 34.7)	20.1 (19.4, 20.8)
P-difference		< 0.001	< 0.001	< 0.001	< 0.001	< 0.001	< 0.001
**Year**							
2013–4	3,594 (12.0)	24.7 (24.3, 25.2)	23.3 (18.1, 29)	1.8 (1.4, 2.2)	32.2 (30.6, 33.9)	41.7 (39.9, 43.4)	24.3 (22.9, 25.8)
2015	3,305 (11.0)	24.2 (23.7, 24.6)	22.4 (17.1, 29)	2.6 (2.1, 3.2)	33.5 (31.8, 35.2)	39.7 (37.9, 41.5)	24.2 (22.6, 25.7)
2016	4,670 (15.5)	24.5 (24.1, 24.9)	22.8 (17.3, 29)	2.5 (2, 2.9)	31.9 (30.5, 33.4)	40.3 (38.8, 41.8)	25.3 (24, 26.6)
2017	8,172 (27.2)	24 (23.7, 24.3)	21.9 (16, 29)	5.0 (4.5, 5.5)	35.1 (33.9, 36.2)	36.1 (34.9, 37.3)	23.8 (22.8, 24.8)
2018	10,299 (34.3)	23.1 (22.8, 23.4)	20.4 (14.5, 28)	8.0 (7.4, 8.6)	38.0 (36.9, 39)	31.8 (30.8, 32.9)	22.1 (21.2, 23)
P-trend		< 0.001	< 0.001	< 0.001	< 0.001	< 0.001	0.002
**Federal district**							
Central	13,906 (46.3)	24.3 (24.1, 24.6)	22.3 (16, 29.7)	4.4 (4, 4.8)	34.3 (33.4, 35.2)	36.1 (35.1, 37)	25.2 (24.3, 26.1)
Far Eastern	1,187 (4.0)	27.2 (26.5, 27.9)	24.8 (19.1, 32.4)	2.0 (1.3, 2.8)	22.9 (20.4, 25.4)	44.0 (41.1, 46.9)	31.1 (28.4, 33.8)
North Caucasian	1,348 (4.5)	19.2 (18.5, 19.8)	16.8 (10.9, 24.3)	14.6 (12.6, 16.6)	45.4 (42.6, 48.2)	25.7 (23.3, 28.1)	14.3 (12.4, 16.2)
Northwestern	4,341 (14.5)	22.5 (22.1, 22.9)	20.3 (14.5, 27.9)	5.4 (4.8, 6.1)	41.1 (39.5, 42.6)	32.1 (30.6, 33.5)	21.4 (20.2, 22.7)
Siberian	4,159 (13.8)	24.2 (23.8, 24.6)	22.3 (16.8, 29)	3.4 (2.8, 3.9)	32.6 (31, 34.1)	40.6 (39, 42.2)	23.5 (22.1, 24.9)
South	1,509 (5.0)	24 (23.3, 24.6)	21.8 (16.9, 28.3)	2.5 (1.7, 3.2)	33.2 (30.7, 35.6)	42.9 (40.4, 45.5)	21.5 (19.4, 23.5)
Ural	1,397 (4.7)	25.1 (24.5, 25.8)	22.3 (15.8, 30.3)	3.7 (2.8, 4.7)	35.02 (32.5, 37.8)	36.4 (33.8, 39)	24.7 (22.4, 27)
Volga	2,193 (7.3)	23.4 (22.8, 23.9)	20.9 (15.5, 29.1)	4.8 (4, 5.7)	36.5 (34.3, 38.6)	36.4 (34.3, 38.6)	22.3 (20.5, 24.1)
P-difference		< 0.001	< 0.001	< 0.001	< 0.001	< 0.001	< 0.001

^a^Values represent age group, sex, season, year of data collection and federal district adjusted means, quantiles or proportions estimated via marginal values following linear regression, quantile regression and multinomial logistic regression for the mean, median and proportions, respectively. Values are fixed at the average level for the population under study apart from sex, which was weighted to represent the Russian population (53.7% female). Values in parentheses are 95% CI except for N and the median, which are the percent of the total sample and inter-quartile range, respectively. Data are from 2013 to 2018 and are from all months of the year.

**Figure 1:**
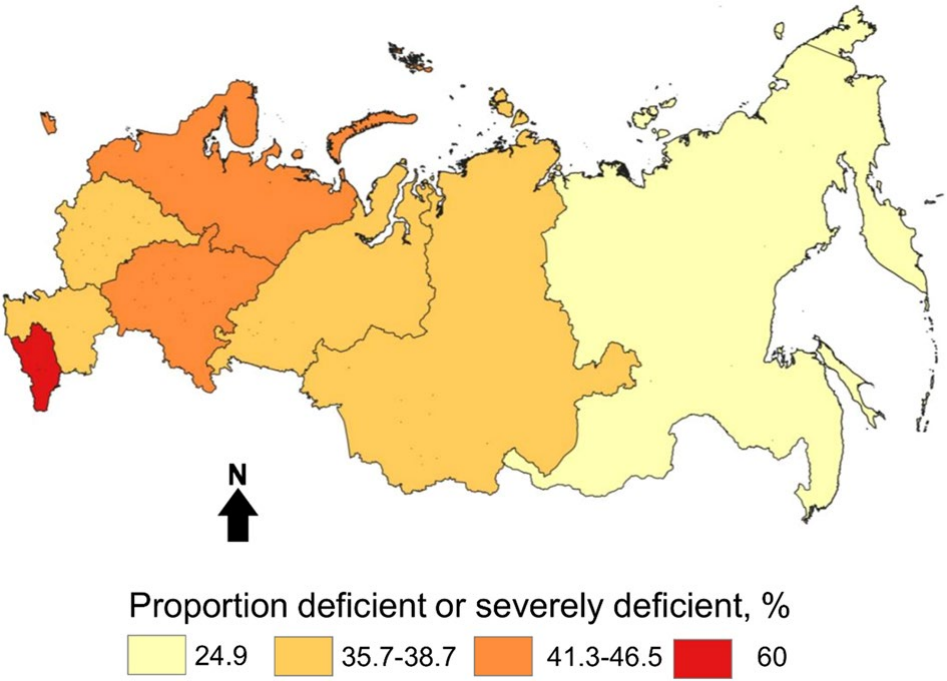
Proportion of sample with severely deficient or deficient 25(OH)D concentrations (< 20 ng/mL) by Federal District. Data are from 2013 to 2018 and are from all months of the year. The map was produced by the authors using QGIS 3.10 (https://www.qgis.org) a free and open-source desktop Geographic Information System application.

Mean serum 25-hydroxyvitamin D (25(OH)D) concentrations across the entire sampled population was 24.1 ng/mL (95% confidence interval [CI] 24.0, 24.3) and the median was 22 ng/mL (interquartile range [IQR]: 16, 29). Just under 6% of the population had severe deficiency defined as < 10 ng/mL (5.6%; 95% CI 5.4, 5.9), 33.8% were deficient (10–19.9 ng/mL) and 36.4% were insufficient (20–29.9 ng/mL) (see Supplemental Fig. S1 for the distribution overall and by season). Only 3.5% of the screened population had concentrations ≥ 50 ng/mL, suggesting low use of high-dose vitamin D supplements, Additional unadjusted results for the total population and stratified by age, sex, season, year and region are provided in Supplemental Table S1. A map showing the proportion of the population with deficient or severely deficient 25(OH)D concentrations is shown as Fig. 1.

In analyses adjusting for sex, season, year and region, mean 25(OH)D concentrations varied by age in a non-linear fashion; they were highest among those 30–44 years but lowest among those 19–29 and ≥ 75 years, respectively (Table 1). About 11% of adults ≥ 75 years had severe deficiency and 11.2% were deficient, but not severely so. Younger adults had the second-highest prevalence of deficient and severely deficient concentrations. 25(OH)D concentrations also varied by sex, with female having somewhat higher concentrations than male, though the difference is likely not clinically meaningful. On the other hand, season was strongly associated with 25(OH)D concentrations, the average and proportion severely deficient and deficient being highest in winter/spring than summer/fall. Nearly half 46% of adults had severe or deficient concentrations of 25(OH)D in the winter/spring compared to 30.3% in the summer/fall. Average 25(OH)D concentrations were lowest in April (21.4 ng/mL) and highest in September (28.0 ng/mL), with the proportion deficient or severely deficient of 53.6% and 22.0%, respectively.

Average 25(OH)D concentrations declined modestly over the study period, but the proportion with severe deficiency increased nearly fourfold (from 1.8% [95% CI 1.4,2,2] in 2013–2014 to 8% [95% CI 7.4, 8.6] in 2018). As this analysis was mutually adjusted for age, sex, season, and region, differences in who was sampled, based on these variables, cannot explain this change. Lastly, average and median 25(OH)D concentrations were highest in the Far Eastern Federal District and lower in the North Caucasian and Northwestern Federal District.

Analyses limited to Moscow city and region where a bulk of the sample resided are shown in Supplemental Table S2 and were generally similar to the analyses of the total population.

Sex-stratified analyses revealed some important differences in the relation between these variables and 25(OH)D concentrations (see Table 2). Average 25(OH)D concentrations were higher among younger female as compared to younger male, but among older adults, the opposite was observed. The difference between mean concentrations by season was much stronger among males than females: + 29% higher in summer/fall compared winter/spring among male and + 12% for female. The decrease in 25(OH)D concentrations over time was observed in both groups but was declined more dramatically among males than females. Lastly, Federal District-level differences in mean concentrations varied by sex. Mean values among females were lowest in the North Caucasian region and highest in the Far Eastern region. For males, the Far Eastern region also had the highest values, but the Northwestern region had the lowest values (values in the North Caucasian region were also low).

**Table 2:** Mean 25(OH)D concentrations by population sub-group, stratified by sex.

	Adjusted^a^ Mean (95% CI)		p-interaction
	Female	Male	
**Age group, years**			
18–29	23.8 (23.2, 24.3)	21.2 (20.2, 22.2)	< 0.001
30–44	25.8 (25.5, 26.1)	23.6 (23.0, 24.2)	
45–59	24.4 (24.1, 24.7)	24.1 (23.5, 24.7)	
60–74	23.6 (23.3, 23.9)	24.7 (23.8, 25.6)	
≥ 75	21.1 (20.6, 21.7)	22.1 (20.5, 23.7)	
P-trend	< 0.001	0.001	
**Season**			
Summer/fall	26.0 (25.7, 26.2)	27.2 (26.6, 27.8)	< 0.001
Winter/spring	23.2 (23.0, 23.4)	21.1 (20.7, 21.6)	
P-difference	< 0.001	< 0.001	
**Year**			
2013–2014	24.8 (24.4, 25.3)	25.9 (24.9, 26.9)	< 0.001
2015	24.3 (23.8, 24.8)	25.4 (24.3, 26.6)	
2016	24.7 (24.3, 25.1)	25.3 (24.4, 26.3)	
2017	24.3 (24.0, 24.5)	24.3 (23.6, 25.1)	
2018	23.9 (23.6, 24.2)	21.0 (20.5, 21.6)	
P-trend	0.001	< 0.001	
**Federal district**			
Central	24.7 (24.4, 24.9)	24.2 (23.7, 24.8)	< 0.001
Far Eastern	27.2 (26.4, 28.0)	29.0 (27.1, 30.9)	
North Caucasian	19.0 (18.3, 19.8)	22.1 (20.2, 24.0)	
Northwestern	24.4 (23.9, 24.8)	19.2 (18.5, 19.8)	
Siberian	24.4 (24.0, 24.8)	25.1 (23.9, 26.2)	
South	23.8 (23.1, 24.5)	27.1 (25.4, 28.8)	
Ural	25.3 (24.6, 26)	26.1 (24.2, 27.9)	
Volga	23.2 (22.6, 23.8)	26.2 (24.8, 27.6)	
P-difference	< 0.001	< 0.001	

Data are from 2013 to 2018 and are from all months of the year. ^a^ Values represent age group, season, year of data collection and federal district adjusted means estimated via marginal values following the fitting of a linear regression model.

### Comparisons to other thresholds

Table 3 shows a comparison of the overall crude proportion of the population below/above specific thresholds from other professional advisory organizations including the United States Institute of Medicine (IOM), the UK Scientific Advisory Committee on Nutrition (SACN), the European Food Safety Authority (EFSA), and others. The UK SACN has the most unique thresholds compared to other organizations (2 categories: < 10 ng/mL indicating deficiency and ≥ 10 ng/mL indicating sufficient concentrations), where 5.6% of the sampled population was deemed to be deficient and 94.3% had sufficient concentrations. For the US IOM, 10.3% of the population was deficient, 29.2% may have inadequate concentrations and 60.6% had concentrations in the sufficient range. Numerous organizations define concentrations < 20 ng/mL to be deficient, which equated to 39.4% of the total population in the present sample.

**Table 3:** Proportion of the population meeting specific thresholds regarding serum 25(OH)D status from selected professional advisory organizations.

Advisory organization	% (95% CI)			
	Severely deficient (< 10 ng/mL), %	Deficient (10–19.9 ng/mL), %	Insufficient (20–29.9 ng/mL), %	Sufficient (30–149.9 ng/mL), %
Russian Society of Endocrinologists^64^	5.6 (5.4, 5.9)	33.8 (33.3, 34.4)	36.4 (35.8, 36.9)	24.2 (23.7, 24.7)
	Deficiency (< 10 ng/mL), %	Sufficiency (≥ 10 ng/mL), %		
UK SACN, 2016^68^	5.6 (5.4, 5.9)	94.3 (94.1, 94.6)		
	Deficiency (< 12 ng/mL), %	May be inadequate for some (12–19.9 ng/mL), %	Sufficient, (≥ 20 ng/mL), %	
Institute of Medicine USA^69^, National osteoporosis society UK, 2014^70^	10.3 (9.9, 10.6)	29.2 (28.7, 29.7)	60.6 (60.0, 61.1)	
	Deficiency (< 10 ng/mL), %	Insufficient (10–19.9, ng/mL), %	Sufficient (≥ 20 ng/mL), %	
The European Society for Clinical and Economic Aspects of Osteoporosis, Osteoarthritis and Musculoskeletal Diseases (ESCEO)^71^	5.6 (5.4, 5.9)	33.8 (33.3, 34.4)	60.6 (60.0, 61.1)	
	Deficient (< 20 ng/mL)	Insufficient (20–29.9 ng/mL), %	Sufficient (≥ 30 ng/mL), %	
International endocrinological society^4^, Spanish Society for Research on Bone and Mineral Metabolism, 2011^72^	39.4 (38.9, 40.0)	36.4 (35.8, 36.9)	24.2 (23.7, 24.7)	
	Deficient (< 20 ng/mL), %	Sufficient (≥ 20 ng/mL), %		
European Food Safety Authority (EFSA)^73^	39.4 (38.9, 40.0)	60.6 (60.0, 61.1)		
	Severely deficient (< 10 ng/mL), %	Deficient (10–19.9 ng/mL), %	Adequate (20–29.9 ng/mL), %	Desirable (≥ 30 ng/mL)
Federal Commission of Nutrition, Switzerland^74^	5.6 (5.4, 5.9)	33.8 (33.3, 34.4)	36.4 (35.8, 36.9)	24.2 (23.7, 24.7)
	Deficient (< 20 ng/mL)	Sufficient for general population (≥ 20 ng/mL), %	Insufficient for older population^a^ (20–29.9 ng/mL), %	Sufficient for older population^a^ (≥ 30 ng/mL), %
International Osteoporosis Foundation (IOF, 2010)^75^	39.4 (38.9, 40.0)	60.6 (60.0, 61.1)	35.1 (34.2, 36.1)	22.4 (21.6, 23.2)

Data are from 2013 to 2018 and are from all months of the year.

^a^Defined as < 60 years by the International Osteoporosis Foundation. Because the IOF thresholds are age-specific, this is the only group where the column percentages will not add to 100%.

### Differences by region

**Figure 2:**
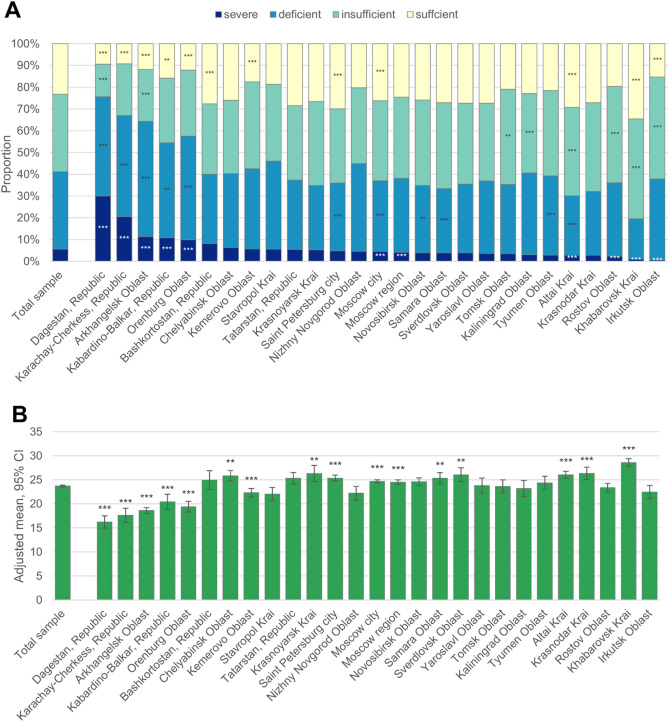
Adjusted proportion with severe, deficient, insufficient and sufficient (**B**) 25(OH)D concentrations and adjusted mean 25(OH)D concentrations (**A**) and by region. Values are fixed at the average level for the population apart from sex, which was weighted to represent the Russian population (53.7% female). Data are from 2013 to 2018 and are from all months of the year. Asterisks indicate p-value compared to total sample: *** p < 0.001; ** 0.001 < p < 0.01.

Figure 2 shows the distribution of 25(OH)D concentrations and mean concentrations by region. Dagestan, Karachay Cherkess, Arkhangelsk, Kabardino Balkaria and Orenburg had mean 25(OH)D concentrations lower than the sample-wide value and had an elevated proportion who had severely deficient and deficient concentrations. Adjusted means were > 25 ng/mL in the following regions: Chelyabinsk, Tatarstan, Krasnoyarsk, Saint Petersburg, Samara, Sverdlovsk, Altai, Krasnodar, and Khabarovsk (p < 0.01 for each comparing to the sample-wide mean).

### Effect of latitude

**Figure 3:**
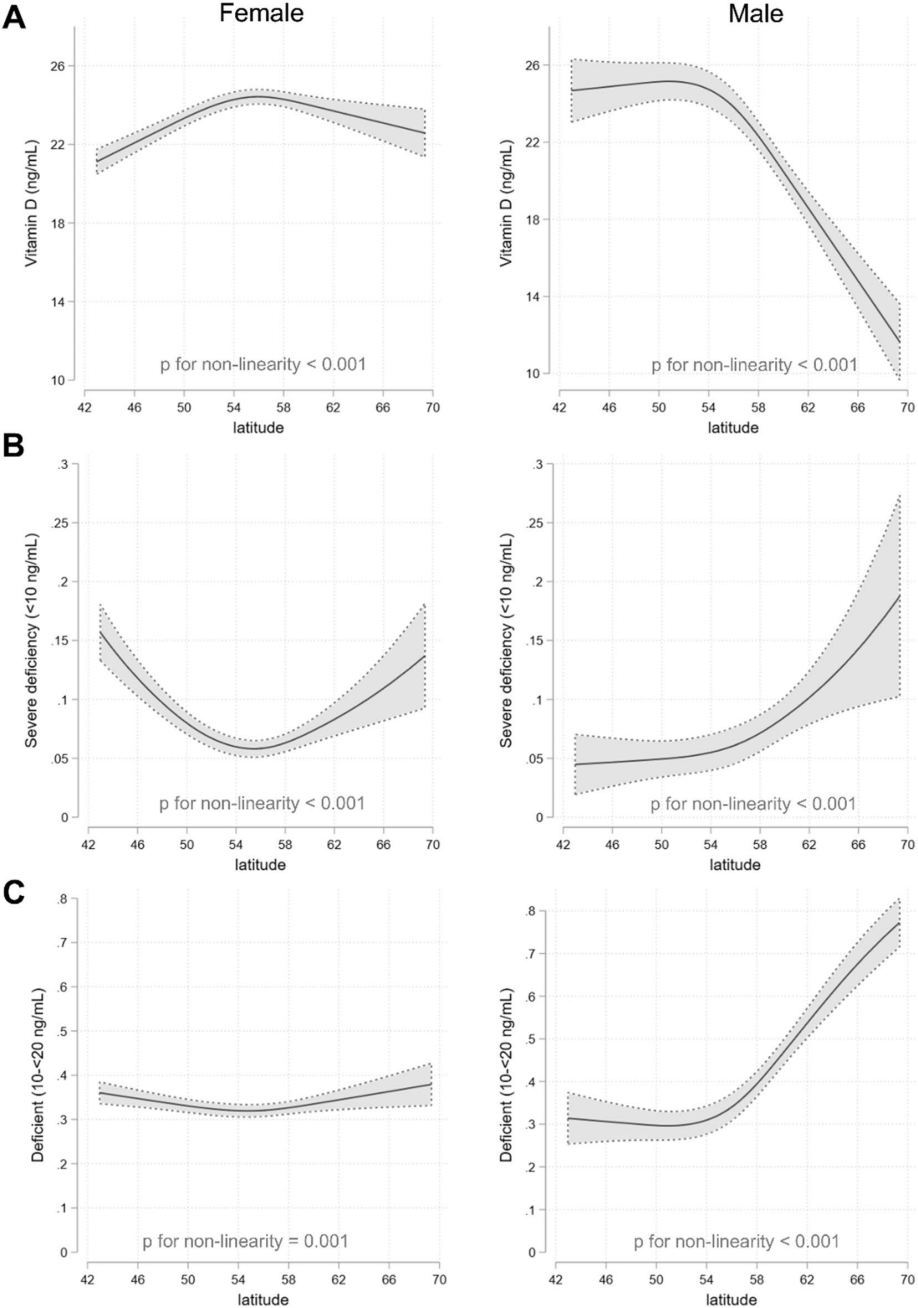
Restricted cubic splines showing association between latitude and mean 25(OH)D concentrations (**A**), proportion with severe deficiency (< 10 ng/mL) (**B**) and proportion with deficiency (10- < 20 ng/mL) (**C**). Shaded gray area represent 95% confidence intervals. Analyses are adjusted for age group, season and year of data collection. Data are from 2013 to 2018 and are from all months of the year. For all outcomes the p-value for interaction between latitude and sex was < 0.001.

In restricted cubic spline analyses differences in the association between latitude and mean 25(OH)D concentrations and the proportion with severely deficient and deficient concentrations were found to differ by sex, in-line with the numerous other variables that appeared to be modified by sex (see Fig. 3). For females, 25(OH)D concentrations were on average lowest at the lowest latitudes and highest at the mid-latitudes (~ 55°N). For reference, the latitude of Moscow is 55.76 and other areas in the 55°N elsewhere in the world include Scotland, Ketchikan, Alaska (USA), or Copenhagen, Denmark. For males, a different relationship was observed where mean concentrations were consistent from the lower-latitudes (~ 42°N) to 55°N, declining precipitously thereafter to ~ 12 ng/mL at ~ 69°N. Similar relationships were observed for the proportion severely deficient, a U-shaped curve for females and a J-shaped curve for males.

## Discussion

To our knowledge, this is the largest assessment of 25(OH)D status in the Russian adult population to-date^27^. In this large cross-sectional study of objectively measured 25(OH)D concentrations in individuals being referred for screening/testing, we found that the prevalence of deficiency among this sample to be 39.7% (4.4% being severely deficient and 35.3% being deficient, but not severely so) according to the local definition provided by the Russian Association of Endocrinologists. Only 23.8% of the population was deemed to have concentrations in the sufficient range (> 30 ng/mL). We also assessed alternative cut points including those of the US IOM, UK SACN, EFSA and others. Except for the UK SACN thresholds, each show that a sizeable proportion of the sample had deficient or insufficient 25(OH)D concentrations (> 30–40%) depending on the specific thresholds being used.

Beyond estimating the proportion of the population with deficient, insufficient and sufficient 25(OH)D concentrations, we also sought to understand how concentrations differed by population sub-group. The observed differences by age, sex, year, federal district, and region may reflect true differences in population-level 25(OH)D concentrations or might alternatively reflect anomalous differences due to selective screening of individuals based on baseline health status, health-seeking behavior and access to healthcare. On the other-hand the seasonal findings likely reflect the actual population-level risk as season is unlikely to impact health seeking behavior or underlying health risk.

Overall, our results (average of 23.9 ng/mL) are generally similar to data for adult population in other European countries with comparable latitudes^6,27–29^. For example: Norway 64° and 69° (26 ± 7.04 [standard deviations] and 28.4 ± 7.8) ng/mL; Sweden 58° (27.48 ± 7.64) ng/mL; Finland 60°-70° (27.08 ± 5.28) ng/mL; Denmark 56° (26 ± 7.68) ng/mL; Ireland 51°-54° (22.56 ± 8.88) ng/mL; Netherlands 52° (23.8 ± 8.68) ng/mL; Belgium 51° (19.72) ng/mL; Germany 47°–55° (20.04 ± 7.24) ng/mL; France 43°–49° (24 ± 8) ng/mL; Estonia 59° in summer (23.36 ± 7.08-female; 24.2 ± 7.4—male) ng/mL and Czech Republic 50° (25 ± 4) ng/mL. None of these countries have the north–south gradient of Russia so it is not possible to determine if the J-shaped and U-shaped relation between latitude and 25(OH)D concentrations would be observed elsewhere. Furthermore, as dietary habits and patterns, especially meat and fish consumption may differ by latitude. As diet is a strong determinant of vitamin D levels, it may be difficult to directly disentangle the effect of latitude on vitamin D status vis-à-vis insolation vs. diet^30,31^. According to standardized data from the ODIN and other studies^6,15,27,28^ for Northern Europe, the prevalence of serum 25(OH)D < 12 ng/mL ranged from 0.4 to 8.4%; our finding is in the middle of this range (4.4%). Prevalence of deficiency (< 20 ng/mL) varies from 6.6 to 33.6% in adults, which is slightly less in comparison with the data observed here (35.3%).

In our data, the most important factor affecting the concentration of 25(OH)D in the blood was seasonality (p < 0.001) consistent with expectations^32,33^. This finding is directly related to an increase in the insolation level in the summer. This pattern was observed in limited Russian samples^34^, as well as studies conducted elsewhere, including Germany^35^, Belgium^36^, Portugal^37^. Compared to some other studies, the seasonal differences observed here were somewhat less dramatic than other studies, potentially due to differences in seasonal definitions or challenges comparing data from random and non-random samples^38,39^. For northern Russian cities, an increase in the concentration of 25(OH)D in the summer can be associated with vacations, often spent in sunny countries with lower latitude. In addition, seasonality should be considered in combination with lifestyle and culture. For example, females in the North Caucasus region tend to have more limited outdoor activities and typically cover their body with longer clothes, which likely contributed to the observed difference between male and female in that region (adjusted mean 22.1 ng/mL for male and 19.0 ng/mL for female), with a greater discrepancy observed in the winter/spring (27.5 ng/mL in summer/fall for male compared to 20.3 ng/mL in winter/spring and 20.7 ng/mL in summer/fall for female compared to 17.8 ng/mL in winter/spring). Wearing of concealing clothing has previously been shown to be associated with lower vitamin D concentrations among females in other countries^40^. Furthermore, throughout the middle east, 25(OH) D status tends to be lower among females as compared to males^41^.

With regards to 25(OH)D concentrations by sex more questions need to be addressed. While most participants in this study were female, we did observe that they on average, had modestly higher concentrations. One explanation for the imbalance of the total sample size is that females in Russia tend to engage in more health-seeking behavior compared to males and are more likely to have regular preventive check-ups. Males tend to be more reactive in their health-seeking behavior and could go to have an analysis only in case of problems with health, which could explain their lower 25(OH)D concentrations^42–44^.

Comparing age differences in 25(OH)D concentrations with studies from other countries we also observe some similarities. In our study, younger adults and older adults had the highest likelihood of having severely deficient values. There also appeared to be less seasonal variation in 25(OH)D concentrations among older adults suggesting that reduced outdoor activity (sun exposure) is a probable explanation and cutaneous synthesis^45^, a finding similar to those observed in other European countries^46^. The finding of lower 25(OH)D concentrations among younger adults is consistent with some studies, including a large study of German adults which found that younger adults were at lower risk of severe deficiency, but greater risk of moderate deficiency^47^. The reason for somewhat lower concentrations among younger adults cannot be elucidated with the present data but could be attributable to differences in dietary patterns or time spent outdoors.

Some important limitations for our data must also be noted. First, the sample includes individuals who sought or were advised to seek vitamin D testing, which is likely to be impacted by numerous factors including baseline health status, healthcare access, propensity to seek healthcare, and other unknown factors. It is unlikely that the data presented here represent a true increase in vitamin D deficiency or differential use of vitamin D testing over time. Notably, the average age of tested individuals declined from 54.1 years in 2013–2014 to 50.2 years, which would provide indirect evidence rejecting a hypothesis that people who were more sick or ill were preferentially tested in later years. There is limited high-quality data to compare to our short-term trend, a large population-based study in Sweden observed no clear secular trend^48^ and a study in Ireland observed an increase in deficiency^49^. However, it is unlikely that health behaviors and diet changed dramatically over this short of a time period to explain the observed fourfold increase in the proportion of individuals with severely deficient concentrations. Unmeasured changes in who and why people were tested for 25(OH)D concentrations is a more likely explanation for this finding.

The over-representation of female and individuals residing in the Moscow area are two examples of this potential bias. In the absence of a population-based nationally representative scientific survey, which can only be undertaken at considerable cost, this data represents the largest collection of vitamin D data for Russia and provides important insights into the possible extent of 25(OH)D deficiency in Russia and identifies some key correlates of 25(OH)D concentrations. For example, past studies of vitamin D concentrations in Russia have lacked data to examine how factors such as region or latitude may impact risk. The lack of data on individual health status, including measurements such as BMI, is a related limitation as health status can affect 25(OH)D concentrations and are not accounted for here. Lastly, additional data on factors such as occupation (a potential proxy for sun exposure), socioeconomic status, dietary intakes, and use of vitamin D supplements would add additional context to the present analysis. Despite these limitations, this study is the largest assessment of vitamin D status of the Russian adult population to-date.

Analysis of vitamin D status in Russia is a complicated task due to its large area (differences in longitude and latitude, climate, amounts of sunny days, ultraviolet index, and other environmental factors), high heterogeneity in ethnicity, culture^50^, lifestyle, and health behaviors. The combination of these factors should be considered when interpreting differences in 25(OH)D concentrations between different geographic areas. In the present analyses we adjusted for available covariates (e.g., age, sex, year), so these factors do not explain differences between places, but other unmeasured factors certainly might.

In addition to geographical location and insolation levels, lifestyle and eating habits also have an influence on 25(OH)D status^51^. For example, Kozlov et al.^50^ compared vitamin D status of two groups of northern indigenous Russian people: (1) Nenets and Komi living in the Arctic (66–67°N) and leading traditional way of life and (2) Urban Komi, Udmurts and Komi-Permiaks living in a non-Arctic area (57–61°N). The authors concluded that the transition from seminomadic to a ‘modernized’ way of life has led to a decrease in the consumption of traditional foods such as venison, reindeer fat, and fish, among the indigenous people of the Russian Arctic. This traditional diet prevents vitamin D hypovitaminosis while the western type of diet tends to correlate with a lower 25(OH)D concentrations in the absence of vitamin D fortification.

Moving into discussion about whether mandatory or voluntary vitamin D fortification of food products can be an effective intervention, insights can be gained from the Northern European experience. The northern European population is considered at risk for vitamin D insufficiency, because of the undetectable cutaneous synthesis of vitamin D at high latitudes during the winter^15^, leaving inhabitants more dependent on dietary sources of vitamin D. Several countries have, therefore, introduced a vitamin D food-fortification policy. In a Norwegian longitudinal study of 2,668 non-smoking individuals, there was a small increase in mean vitamin D concentrations between 1994 and 2008 (from 21.5 to 22.2 ng/mL)^10^. A larger increase in mean 25(OH)D concentrations was observed in a Finnish cross-sectional study of 10,185 individuals: from 19.2 ng/mL in 2000 to 26.0 ng/mL in 2011^52^ and is mainly explained by food fortification, especially of fluid milk products and increased vitamin D supplement use. Other studies have corroborated that 25(OH)D concentrations have increased dramatically in Finland^53^. In Sweden, a large cross-sectional study between 1986 and 2014, based on data from seven population-based surveys, indicated no clear upward or downward trend of 25(OH)D concentrations^48^. From countries outside of northern Europe, some have experienced an increase (e.g., Canada^54^, the USA^55^ and Ireland^56^), while others have experienced a decrease (e.g., the USA^57^, Greenland^58^) in 25(OH)D concentrations over time. At the moment, there is no mandatory food fortification policy in the Russian Federation and a low presence of vitamin D fortified dairy products in the market, so there is a potential opportunity to improve population status by implementing food fortification initiatives.

It also seems that the COVID-19 pandemic, which came to Russia at the beginning of 2020 is changing consumer’s attitude to fortified products and in particular promotion of vitamin D supplementation which has led to a dramatic increase of vitamin D sales. Due to recent high-profile reports, correcting vitamin D deficiency might help prevent COVID-19 illness and help limit complications, though the true value of vitamin D supplementation for COVID prevention remains unclear, though observational studies and limited trials suggest a potential benefit^59–62^.

## Conclusion

In this large study, using routinely collected laboratory assessments of vitamin D status, we observed that the proportion of the sample with severely deficient concentrations was 4.4% and that a third of the population had deficient, but not severely so concentrations of vitamin D. Some interesting differences in 25(OH)D concentrations by personal characteristics and geography were observed, including a non-linear relationship with latitude, dramatic geographic differences and differing patterns by sex. Nationally-representative and randomly sampled data are needed to quantify the burden of 25(OH)D deficiency, but such studies are very resource intensive. Secondary analyses of existing data can help fill data gaps, inform future studies and identify patterns and differences in 25(OH)D concentrations, meriting more detailed investigation.

## Methods

### Ethics declaration

This study is a secondary analysis using data or biospecimens not collected specifically for this study. All subjects provided written informed consent for their de-identified data to be used in secondary analyses. The data were provided without identifiable information by someone without any role in this research study except providing said de-identified data. Therefore, this study is not considered human subjects research and no additional approvals (e.g., Institutional Review Board approval) were required. All research was performed in accordance with relevant guidelines/regulations and have performed in accordance with the Declaration of Helsinki.

### Data description

Data on serum 25-hydroxyvitamin D (25(OH)D) concentrations from adults with single visits ≥ 18 years were collected from January 1, 2013 through September 30, 2018 from InVitro laboratories, a commercial Russian laboratory. There were no specific inclusion/exclusion criteria, and no information exists on reasons why those individuals decided to analyze vitamin D and if there were special concerns or chronic diseases. The de-identified data set included the following variables: federal district, region, town, sex, age, month and year the sample was taken and 25(OH)D concentrations. The term “region” was used to broadly refer to official federal subjects of the Russian Federation which may be referred to as oblasts, republics, krais, autonomous okrugs, federal cities and autonomous oblasts, but are hereafter referred to as regions for simplicity. The final sample size was 30,040 and no subjects had missing data for any variable of interest.

### Outcomes: biochemical analysis and parameterization

Fasting venous blood samples were drawn in the morning and sent to the laboratory the same day at temperature + 2—+ 8 °C. Blood analyses were performed by InVitro laboratories. Quantitative analysis of 25(OH)D in serum was done by chemiluminescent microparticle immunoassay (Architect i2000, Abbot, USA). In the published literature, the intra-assay coefficient of variation is estimated to be in the range of 2.16–4.6% and the inter-assay variability is estimated to be 2.74–4.74%^63^. Laboratory-specific CVs were not available. All participants signed an informed consent form.

25(OH)D status was also initially assessed using five classification stages: severe deficiency (< 10); deficiency (10–< 20 ng/mL); insufficiency (20–< 30 ng/mL), sufficient status (30–< 150 ng/mL), and toxicity (≥ 150 ng/mL). This classification is based on clinical recommendations of the Russian Association of Endocrinologists^64^. For analyses of 25(OH)D status categories, the very small number of participants with values in the toxicity range (n = 15) were excluded (they were included in all other analyses). Additional analyses examined the overall prevalence of deficient and sufficient 25(OH)D concentrations using alternative criteria (e.g., United States Institute of Medicine, UK SACN, The European Society for Clinical and Economic Aspects of Osteoporosis, Osteoarthritis and Musculoskeletal Diseases (ESCEO).

### Exposure variables

Age was grouped into 5 levels: 18–29, 30–44, 45–59, 60–74, ≥ 75 and season was grouped into two variables (winter/spring [November–May] and summer/fall [June-October]) based on insolation levels in Russia. Because a limited sample size in 2013, data from 2013 to 2014 was combined. For regional analyses, we focused on regions with ≥ 200 subjects to ensure results were statistically stable. Latitude was obtained from a combination of geographic centroids of towns and regions obtained from Google Maps.

### Statistical analysis

Descriptive statistics, including the mean, median, inter-quartile range and the proportion of sampled individuals with severe, deficient, insufficient, and sufficient values was calculated, along with corresponding 95% confidence intervals where appropriate. The cumulative distribution function was plotted overall and by season. Multivariable analyses were conducted that estimated the covariate-adjusted mean, median and inter-quartile range, and the proportions using linear regression, quantile regression and multinomial logistic regression, respectively. The marginal mean, percentile and proportions were estimated at the average covariate level for the population assuming the same sex distribution of Russia (53.7%). Because many, but not all, previous large-scale descriptive studies have found 25(OH)D concentrations to vary by sex, additional analyses stratified the sub-group specific results by sex for mean vitamin D concentrations and the statistical interaction by sex was formally tested^65,66^. Multivariable-adjusted regional analyses were conducted for regions with ≥ 200 subjects (N = 27 regions; 88.6% of sampled population) examining the distribution of 25(OH)D concentrations and the mean 25(OH)D concentrations. For each region a p-value was calculated by comparing the proportion or mean to the overall population value. Further, as a large proportion of the sample came from the Moscow area, additional Moscow-only analyses were conducted using the same methods as for the overall sample. To assess the relationship between latitude and 25(OH)D concentrations, restricted cubic splines were used (3 knots) for the mean and the proportion severely deficient and deficient using linear and logistic regression models^64,67^. These analyses were stratified sex and non-linearity was assessed using the likelihood ratio test. Data analysis was carried out using Stata 16.0 (College Station, TX, 1985–2019). Because of the large sample size and the number of statistical tests being conducted all tests were executed at a two-sided α-level of 0.01.

## Supplementary Information

Supplementary Information.

## Data Availability

The datasets generated during the current study are available from the corresponding author on request.
