# Supplementary material for: Vitamin D status of the Russian adult population from 2013 to 2018
Source: Sci Rep. 2022 Oct 5;12:16604. doi: 10.1038/s41598-022-21221-4 (PMC9533264; doi:10.1038/s41598-022-21221-4)
Supplement: Supplementary file 1 — Supplementary Information. [file 41598_2022_21221_MOESM1_ESM.docx]

**Supplemental Figures & Tables**

**Vitamin D status of the Russian adult population: 2013-2018**

**Figure 1.** Cumulative distribution of 25(OH)D concentrations with reference lines for severe deficiency, deficiency and insufficiency overall and stratified by season

**
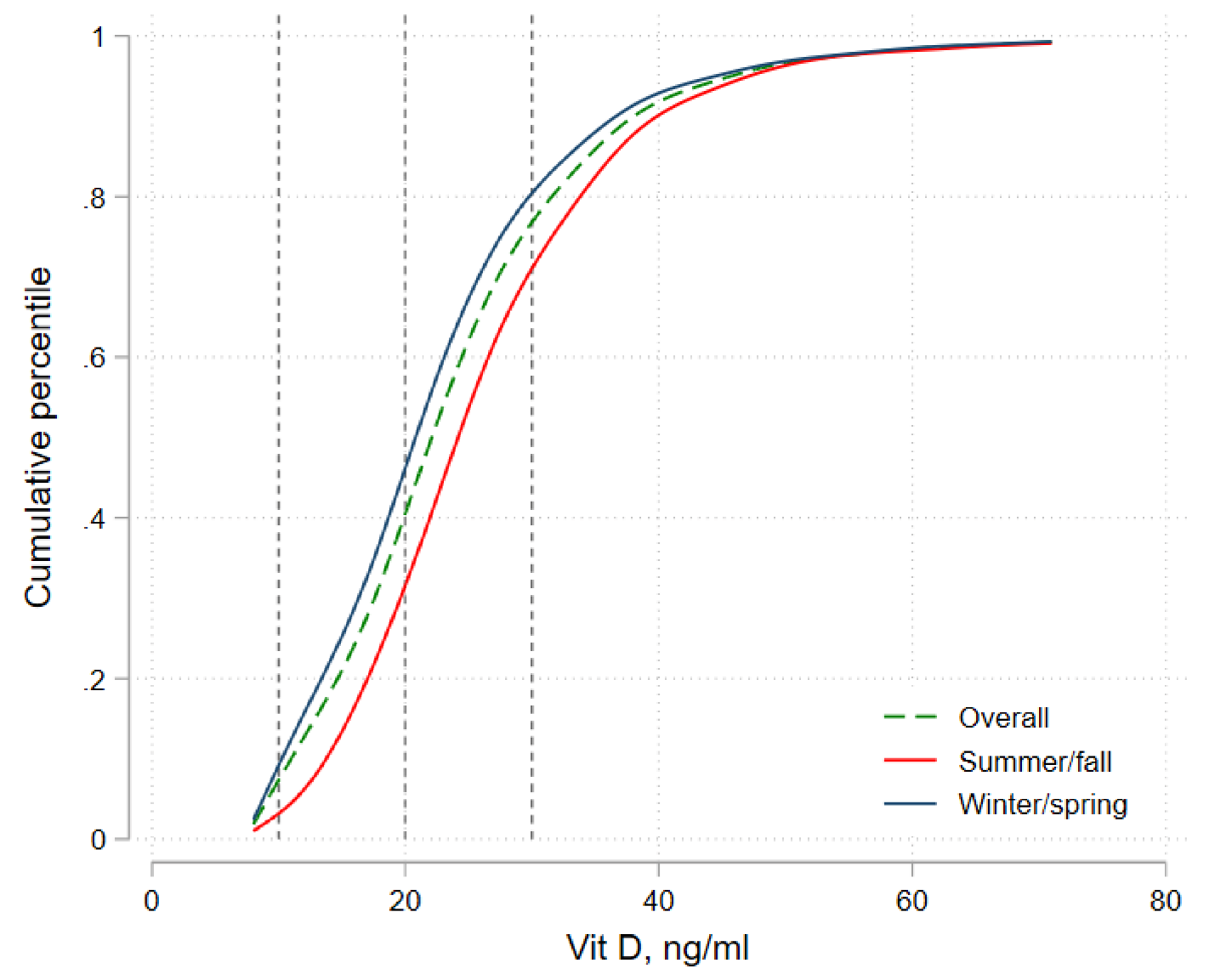
**

**Table S1.** Population characteristics and average, median, and proportion with severely deficient, deficient, insufficient and sufficient 25(OH)D concentrations

|  | n | Mean, ng/mL | Median ng/mL (IQR) | Severe deficiency (<10 ng/mL), % (95% CI) | Deficiency (10-19.9 ng/mL), % (95% CI) | Insufficiency (20-29.9 ng/mL), % (95% CI) | Sufficient (30-149.9 ng/mL), % (95% CI) |
| --- | --- | --- | --- | --- | --- | --- | --- |
|  |  |  |  |  |  |  |  |
| Total | 30,040 | 24.1  (24.0, 24.3) | 22  (16, 29) | 5.6  (5.4, 5.9) | 33.8  (33.3, 34.4) | 36.4  (35.8, 36.9) | 24.2  (23.7, 24.7) |
|  |  |  |  |  |  |  |  |
| Age group, y |  |  |  |  |  |  |  |
| 18-29 | 2,478 | 23.2  (22.7, 23.7) | 21  (15, 28) | 7.8  (6.8, 9) | 35.9  (34, 37.8) | 34.0  (32.2, 35.9) | 22.2  (20.6, 23.9) |
| 30-44 | 7,520 | 25.2  (24.9, 25.6) | 22  (16.8, 30.1) | 4.7  (4.2, 5.2) | 32.3  (31.2, 33.3) | 36  (34.9, 37.1) | 27.1  (26.1, 28.1) |
| 45-59 | 9,936 | 24.3  (24.1, 24.5) | 22  (16.6, 29) | 4.9  (4.5, 5.4) | 32.3  (31.3, 33.2) | 38.4  (37.5, 39.4) | 24.4  (23.5, 25.2) |
| 60-74 | 7,990 | 23.9  (23.6, 24.1) | 22  (16, 29) | 5.1  (4.6, 5.6) | 34.8  (33.7, 35.8) | 36.7  (35.7, 37.8) | 23.4  (22.5, 24.3) |
| ≥75 | 2,116 | 21.4  (20.9, 21.9) | 19  (13, 26) | 11.6  (10.3, 13.1) | 40.8  (38.7, 42.9) | 29.1  (27.2, 31.1) | 18.5  (16.9, 20.2) |
|  |  |  |  |  |  |  |  |
| Sex |  |  |  |  |  |  |  |
| Female | 24,931 | 24.3  (24.1, 24.5) | 22  (16, 29) | 5.4  (5.1, 5.7) | 33.0  (32.5, 33.6) | 37  (36.4, 37.6) | 24.6  (24, 25.1) |
| Male | 5,109 | 23.2  (22.9, 23.6) | 21  (15, 29) | 6.8  (6.1, 7.5) | 37.7  (36.4, 39.1) | 33.2  (31.9, 34.5) | 22.3  (21.2, 23.5) |
|  |  |  |  |  |  |  |  |
| Season |  |  |  |  |  |  |  |
| summer/fall | 11,543 | 26.2  (26, 26.4) | 24  (18, 31) | 3.2  (2.9, 3.6) | 26.5  (25.7, 27.3) | 40  (39.1, 40.9) | 30.3  (29.4, 31.1) |
| winter/spring | 18,497 | 22.8  (22.6, 23) | 20.9  (15, 28) | 7.1  (6.8, 7.5) | 38.4  (37.7, 39.1) | 34.1  (33.4, 34.8) | 20.4  (19.8, 21) |
|  |  |  |  |  |  |  |  |
| Year |  |  |  |  |  |  |  |
| 2013-4 | 3,594 | 25  (24.6, 25.3) | 23.3  (17.9, 30) | 2.2  (1.7, 2.7) | 31.5  (30, 33) | 41.3  (39.7, 42.9) | 25.1  (23.7, 26.5) |
| 2015 | 3,305 | 24.7  (24.3, 25.1) | 23  (17, 30) | 2.9  (2.4, 3.6) | 31.7  (30.1, 33.3) | 40  (38.3, 41.6) | 25.4  (24, 26.9) |
| 2016 | 4,670 | 24.9  (24.6, 25.3) | 23  (17, 30) | 2.8  (2.3, 3.3) | 30.3  (29, 31.7) | 40.7  (39.3, 42.1) | 26.2  (25, 27.5) |
| 2017 | 8,172 | 24.4  (24.1, 24.7) | 22  (16, 29) | 5.3  (4.9, 5.8) | 33.2  (32.2, 34.2) | 36.7  (35.7, 37.8) | 24.8  (23.9, 25.7) |
| 2018 | 10,299 | 23  (22.8, 23.3) | 20  (14, 28) | 9.2  (8.7, 9.8) | 37.5  (36.5, 38.4) | 31.2  (30.3, 32.1) | 22.1  (21.3, 22.9) |
|  |  |  |  |  |  |  |  |
| Federal district |  |  |  |  |  |  |  |
| Central | 14,929 | 24.6  (24.4, 24.8) | 22.1  (16, 30) | 5.0  (4.6, 5.3) | 32.8  (32.1, 33.6) | 36.6  (35.8, 37.4) | 25.6  (24.9, 26.4) |
| Far Eastern | 2,153 | 27.6  (26.8, 28.4) | 25  (20, 32) | 2.3  (1.6, 3.3) | 21.7  (19.5, 24.2) | 44.3  (41.5, 47.2) | 31.7  (29.1, 34.4) |
| North Caucasian | 1,539 | 19.4  (18.8, 20) | 17  (11, 25) | 17.1  (15.2, 19.2) | 42.2  (39.6, 44.9) | 25.7  (23.5, 28.1) | 14.9  (13.1, 16.9) |
| Northwestern | 4,593 | 22.4  (22.1, 22.8) | 20  (14, 28) | 7.5  (6.8, 8.4) | 40.6  (39.1, 42.1) | 30.5  (29.1, 31.9) | 21.4  (20.2, 22.6) |
| Siberian | 4,545 | 24.6  (24.2, 24.9) | 23  (17, 29) | 3.9  (3.3, 4.5) | 31  (29.6, 32.4) | 41  (39.5, 42.5) | 24.2  (22.9, 25.5) |
| South | 1,642 | 24.5  (23.9, 25.1) | 22  (17.4, 29) | 2.9  (2.1, 3.8) | 31.2  (28.9, 33.6) | 43.4  (40.9, 45.9) | 22.5  (20.5, 24.7) |
| Ural | 1,566 | 25.4  (24.5, 26.2) | 22.1  (16, 30) | 4.9  (3.9, 6.1) | 34.1  (31.6, 36.6) | 35.9  (33.4, 38.4) | 25.2  (23, 27.5) |
| Volga | 2,442 | 23.6  (23, 24.1) | 21  (15, 29) | 6.5  (5.6, 7.6) | 35.2  (33.2, 37.2) | 35.5  (33.6, 37.6) | 22.7  (21, 24.5) |

Value in parentheses is 95% confidence interval for mean and proportions and inter-quartile range for the median.

**Table S2.** Multivariable adjusted^a^ 25(OH)D status overall and by population sub-group in Moscow city and Moscow region (n=12,426) 2013-2018

|  | Mean, ng/mL | Median ng/mL (IQR) | Severe deficiency (<10 ng/mL), % (95% CI) | Deficiency (10-19.9 ng/mL), % (95% CI) | Insufficiency (20-29.9 ng/mL), % (95% CI) | Sufficient (30-149.9 ng/mL), % (95% CI) |
| --- | --- | --- | --- | --- | --- | --- |
| Total | 24.5  (24.2, 24.8) | 22.3  (16.5, 29.6) | 4.7  (4.4, 5) | 35.5  (34.8, 36.2) | 36.1  (35.4, 36.8) | 23.6  (23, 24.3) |
|  |  |  |  |  |  |  |
| Age group, y |  |  |  |  |  |  |
| 18-29 | 23.4  (22.6, 24.2) | 20.9  (15.2, 28.6) | 6.8  (5.8, 7.8) | 37.6  (35.6, 39.6) | 33.8  (31.9, 35.7) | 21.7  (20.1, 23.4) |
| 30-44 | 26.1  (25.6, 26.6) | 23.9  (17.7, 31.6) | 3.7  (3.3, 4.2) | 33.1  (32, 34.3) | 36.3  (35.2, 37.5) | 26.8  (25.7, 27.9) |
| 45-59 | 24.8  (24.3, 25.2) | 22.9  (17.2, 29.6) | 4.1  (3.7, 4.5) | 33.6  (32.6, 34.7) | 38.4  (37.3, 39.4) | 23.9  (23, 24.8) |
| 60-74 | 24.2  (23.8, 24.7) | 21.9  (16.2, 29.1) | 4.7  (4.2, 5.2) | 36.9  (35.7, 38.1) | 35.8  (34.6, 36.9) | 22.6  (21.6, 23.6) |
| ≥75 | 21.6  (20.9, 22.4) | 18.9  (13.2, 26.6) | 11.8  (10.3, 13.4) | 43.5  (41.2, 45.7) | 27.3  (25.4, 29.3) | 17.3  (15.7, 19) |
| P-trend | <0.001 | <0.001 | <0.001 | <0.001 | 0.002 | <0.001 |
|  |  |  |  |  |  |  |
| Sex |  |  |  |  |  |  |
| Female | 24.7  (24.5, 25) | 22.7  (22.5, 23.0) | 4.2  (3.8, 4.6) | 32.5  (31.6, 33.4) | 37.4  (36.4, 38.3) | 25.9  (25.1, 26.8) |
| Male | 24.3  (23.7, 24.9) | 21.7  (21.2, 22.3) | 4.8  (3.8, 5.7) | 34.2  (31.9, 36.4) | 36  (33.8, 38.2) | 25.1  (23.1, 27.1) |
| P-difference | 0.21 | 0.002 | 0.28 | 0.20 | 0.28 | 0.46 |
|  |  |  |  |  |  |  |
| Season |  |  |  |  |  |  |
| summer/fall | 26.3  (25.9, 26.8) | 24.1  (18.3, 31.8) | 3.2  (2.6, 3.7) | 27.1  (25.6, 28.5) | 37.8  (36.2, 39.4) | 31.9  (30.4, 33.5) |
| winter/spring | 23.4  (23.1, 23.8) | 21.1  (15.3, 28.3) | 5.4  (4.7, 6) | 37.1  (35.8, 38.5) | 35.5  (34.2, 36.9) | 22  (20.8, 23.1) |
| P-difference | <0.001 | <0.001 | <0.001 | <0.001 | 0.018 | <0.001 |
|  |  |  |  |  |  |  |
| Year |  |  |  |  |  |  |
| 2013-4 | 24.9  (24.4, 25.5) | 23.2  (18, 29.6) | 2.4  (1.7, 3) | 31.6  (29.5, 33.7) | 41.6  (39.4, 43.9) | 24.4  (22.5, 26.4) |
| 2015 | 24.5  (23.9, 25.1) | 22.1  (17.2, 29.6) | 3.3  (2.4, 4.1) | 33.4  (31.1, 35.7) | 38.4  (36, 40.7) | 25  (22.9, 27.1) |
| 2016 | 24.5  (23.9, 25) | 22.1  (17.2, 29.1) | 3.2  (2.4, 4) | 32.8  (30.6, 34.9) | 38.4  (36.2, 40.7) | 25.6  (23.6, 27.6) |
| 2017 | 24.4  (23.9, 24.9) | 22.1  (15.7, 29.6) | 6.1  (5.1, 7) | 33.8  (31.9, 35.6) | 34.9  (33.1, 36.8) | 25.3  (23.6, 27) |
| 2018 | 24.5  (24, 25) | 22.1  (15.2, 30.1) | 7.5  (6.4, 8.6) | 33.5  (31.6, 35.3) | 32.7  (30.9, 34.6) | 26.3  (24.5, 28) |
| P-trend | 0.22 | <0.001 | <0.001 | 0.23 | <0.001 | 0.12 |

^a^ Values represent age group, sex, season and year of data collection adjusted means, quantiles or proportions estimated via marginal values following linear regression, quantile regression and multinomial logistic regression for the mean, median and proportions, respectively. Values are fixed at the average level for the population apart from sex, which was weighted to represent the Russian population (53.7% female).
